# Flexible Programming of Cell-Free Protein Synthesis Using Magnetic Bead-Immobilized Plasmids

**DOI:** 10.1371/journal.pone.0034429

**Published:** 2012-03-28

**Authors:** Ka-Young Lee, Kyung-Ho Lee, Ji-Woong Park, Dong-Myung Kim

**Affiliations:** 1 Department of Fine Chemical Engineering and Applied Chemistry, Chungnam National University, Daejeon, Korea; 2 Department of Materials Science and Engineering, Gwangju Institute of Science and Technology, Gwangju, Korea; University of Connecticut, United States of America

## Abstract

The use of magnetic bead-immobilized DNA as movable template for cell-free protein synthesis has been investigated. Magnetic microbeads containing chemically conjugated plasmids were used to direct cell-free protein synthesis, so that protein generation could be readily programmed, reset and reprogrammed. Protein synthesis by using this approach could be ON/OFF-controlled through repeated addition and removal of the microbead-conjugated DNA and employed in sequential expression of different genes in a same reaction mixture. Since the incubation periods of individual template plasmids are freely controllable, relative expression levels of multiple proteins can be tuned to desired levels. We expect that the presented results will find wide application to the flexible design and execution of synthetic pathways in cell-free chassis.

## Introduction

Applications of cell-free protein synthesis systems have continued to expand beyond the laboratory scale protein preparation to a variety of fields including preparative protein production, *in vitro* protein engineering, *in situ* protein labeling and protein microarrays [1], [2], [3], [4]. Compared to cell-based expression methods, cell-free systems have the unique advantage of unlimited acces sibility to the molecular processes involved. This feature enables mix-and-match applications of various biological or non-biological components for the synthesis of functional proteins and it minimizes complications associated with interactions with existing endogenous cellular pathways. Cell-free synthesis systems also can be readily quantified, standardized and modularized to provide attractive platforms for studying synthetic biology.

ON/OFF control of gene expression is one of the most basic features required for the operation of synthetic gene circuits. Whereas the manipulation of gene expression within the environments of living cells demands sophisticated regulatory networks of effectors, the open nature of cell-free systems offers more direct and facile options to govern ON/OFF gene expression. For example, we have previously described reversible regulation of gene expression using antisense oligodeoxynucleotides (ODNs) in a cell-free protein synthesis system [5]. In this effort, either complete shut down or controlled reduction of gene expression levels was achieved through antisense ODN-mediated degradation of mRNA in the reaction mixture. In addition, ODN-mediated repression of protein synthesis could be reversed by using an anti-antisense ODN sequence that removes antisense ODN from the target mRNA sequence. As a consequence, alternating additions of the antisense and anti-antisense ODNs led to stop-and-go control of protein synthesis. However, this approach requires the repetitive use of large amounts of oligonucleotides. RNase H-dependent mRNA degradation is also not an efficient method with respect to bioenergetics because it leads to futile consumption of resources in the reaction mixture. Moreover, as discussed by Park *et al.* [6], designing an optimal antisense ODN sequence is not always a trivial issue because activation of RNase H is known to depend on nucleotide sequences in the hybridized region of mRNA-DNA complex.

In the study described below, we explored the use of magnetic bead-immobilized plasmids in reversible and programmable cell-free protein synthesis. Following careful optimization of the plasmid-microbead conjugation process, studies were carried out to determine whether the microbead-conjugated plasmid was able to direct efficient protein synthesis and to be readily removed from the reaction mixture to turn off the protein synthesis reaction. Observations made in this effort showed that repeated addition and removal of the microbead-conjugated DNA led to step-wise, repetitive, ON/OFF control of gene expression. The on-demand, reversible programming of cell-free synthesis provides greater flexibility and controllability to processes employed for multiple protein expression. By employing the magnetic microbead-DNA conjugates, we have been able to express a series of proteins through sequential incubation of the microbeads. Furthermore, the relative expression levels of proteins during the sequential expression process can also be manipulated by controlling time periods used to incubate individual magnetic bead-plasmid conjugates.

We anticipate that the results coming from this study will demonstrate the capability of simple writing, erasing and editing of programs for protein synthesis in cell-free systems and their applicability to the design, analysis and evaluation of synthetic gene circuits.

## Results and Discussion

### Cell-free protein synthesis using microbead-conjugated plasmids

**Figure 1:**
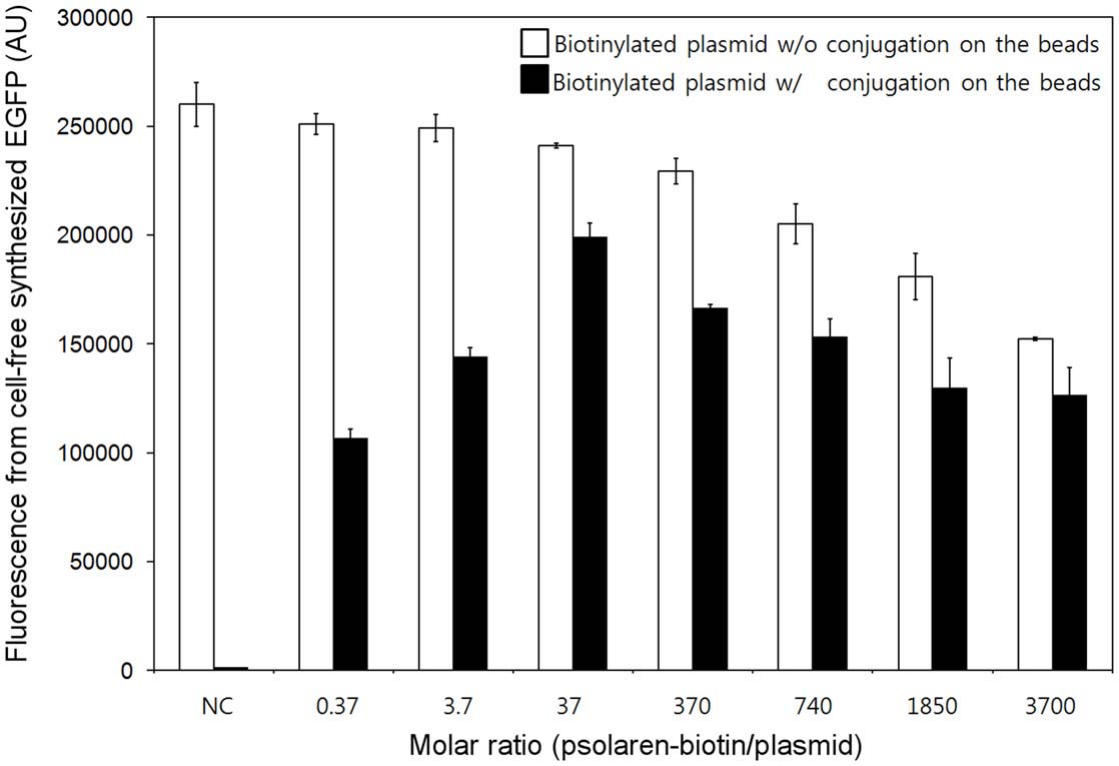
Effect of the psoralen-biotin to plasmid ratio on the expression efficiency of the treated plasmids. Negative control (NC) with no biotinylation of pK7EGFP. 4 pmol of the pK7EGFP was treated with varying concentrations of psoralen-biotin, followed by cell-free expression with (filled bars) or without (blank bars) conjugation on the streaptavidin-coated magnetic microbeads. The amount of the cell-free synthesized EGFP was determined by measuring fluorescence after 3 h incubation of the reaction mixture at 30°C.

In this study we have explored the use of immobilized DNA as a template for reversible programming of cell-free protein synthesis. For this purpose, plasmid DNA was tethered to the surface of streptavidin-coated magnetic microbeads using a psoralen-biotin linker as described in the Material and Methods section. While the efficiency of plasmid-bead conjugation process should be directly proportional to the concentration of the psoralen-biotin, high concentrations of this substance would also increase the probabilities of chemical modifications of bases in the ORF and regulatory sequences of the target gene, resulting in less efficient protein synthesis. Consequently, we first determined the ratio of the template plasmid and psoralen-biotin that would lead to an optimum efficiency for both immobilization and gene expression. The plasmid pK7EGFP (10 µg, 4 pmol) was treated with varying amounts of psoralen-biotin (1.48, 14.8, 148, 2960, 7400, 14800 pmol) , and incubated in a standard reaction mixture required for cell-free protein synthesis in the presence and absence of the conjugation process with 250 µg of strepatavidin microbeads. As expected, treatment of the plasmid with increasing concentrations of psoralen-biotin led to correspondingly lower efficiencies of protein synthesis based on the EGFP fluorescence in the cell-free protein synthesis reaction mixture (Figure 1). However, an optimal 1∶37 ratio of plasmid to psoralen-biotin was found to exist for maximal protein synthesis when the treated plasmid was conjugated on the microbeads, most likely a result of competition between damage on the nucleotide and biotin-mediated retention of plasmid on the surface of the microbeads. The microbead-plasmid conjugate prepared using a ratio of plasmid to psoralen to microbead of 1 pmol∶ 37 pmol∶ 125 µg was observed to yield *ca.* 80% the amount of target protein in comparison to a control reaction using the same amount of unmodified plasmid. The optimal ratio (1∶37∶125) did not vary significantly even when the expression vector for the target protein was changed, and the conjugate prepared in this ratio was used in subsequent experiments.

### Utilization of the magnetic bead-conjugated plasmid as a reusable template for cell-free protein synthesis

**Figure 2:**
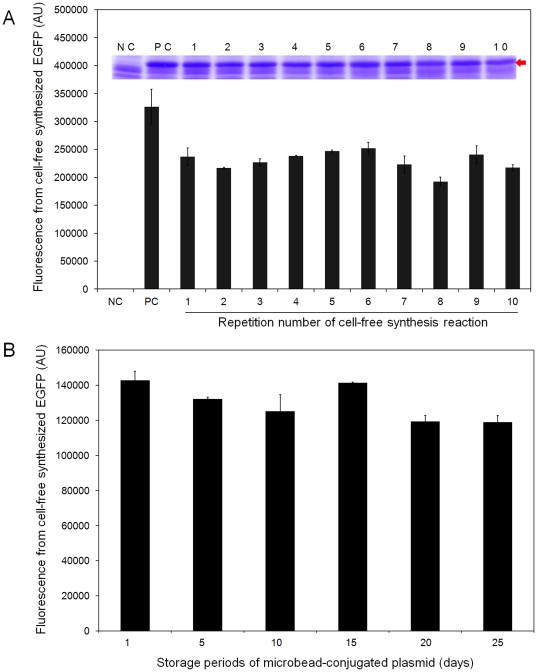
Stable maintenance of the functional integrity of the microbead-conjugated plasmid during repeated synthesis reactions and long-term storage. pK7EGFP, not immobilized to microbeads, was used as a positive control (PC) and the negative control (NC) involved the cell-free protein synthesis mixture not containing DNA. (A) Magnetic microbead-conjugated plasmid (pK7EGFP) was repeatedly used for cell-free synthesis of EGFP as described in the Materials and Methods section. The size and relative amounts of the synthesized EGFP from each round of reaction were analyzed by using SDS-PAGE gel analysis with Coomassie blue staining. The arrow indicates the expected size of EGFP. (B) Magnetic microbead-conjugated plasmid (pK7EGFP) was used for cell-free synthesis of EGFP after being stored at 4°C for the indicated days. Three independent preparations of the microbead-conjugated plasmid were used in this experiment to obtain the average values and standard deviations.

We expected that the template plasmid immobilized on the magnetic beads would serve as a recyclable component of protein synthesis. The data in Figure 2A arise from studies probing protein expression following repeated wash and re-incubation cycles using the plasmid pK7EGFP-conjugated, magnetic microbeads. In these cases, the progress of protein synthesis was monitored using EGFP fluorescence analyses of reaction mixtures and band intensity analyses of the expressed proteins on Coomassie blue-stained SDS-PAGE gels. The results demonstrate that the microbead-immobilized plasmid directs protein synthesis with almost constant yields over repeated reaction cycles. In addition, observations made in this study show that the microbead-conjugated plasmid can be stored for several weeks without displaying decreased capacity for protein synthesis (Figure 2B). Consequently, it appears that bead-conjugated plasmids can be used as recyclable off-the-shelf reagents for protein generation, thus, eliminating the need to prepare fresh template DNA for each protein synthesis batch.

### ON/OFF control of gene expression by addition and removal of the plasmid-conjugated magnetic microbeads

**Figure 3:**
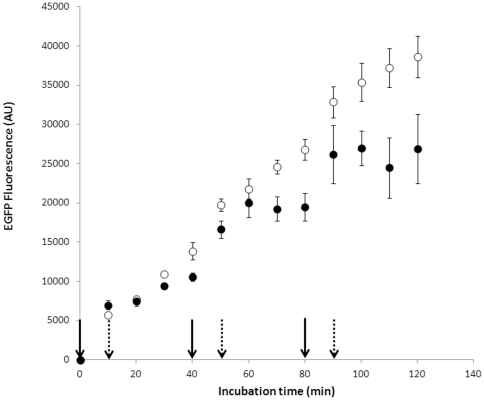
Repeated ON/OFF control of cell-free protein synthesis. EGFP was expressed from microbead-conjugated plasmid in the reaction mixture employing the extract with (BL21(DE3), filled circles) or without (BL21(DE3)Star™, open circles) RNase E activity. The plasmid-conjugated microbeads were incubated for 10 min and then removed from the cell-free protein synthesis reaction mixture. The reaction mixture not containing the microbead was incubated for 30 min. This process was repeated 3 times. Bold arrows and dotted arrows indicate the time points for addition and removal of the plasmid-conjugated microbeads, respectively.

Based on the finding that the microbead-conjugated plasmid serves as a recyclable template for cell-free protein synthesis, we explored a method for ON/OFF control of protein expression by using repeated addition and removal of the magnetic microbead-conjugated plasmid from the reaction mixture. Cycles involving incubation, recovery and regeneration of the plasmid-magnetic microbead conjugate were repeated while carrying out EGFP fluorescence analyses of the reaction mixture. As the data in in Figure 3 show, the EGFP fluorescence from the reaction mixture displays a stepwise increase in response to the removal and re-addition of the microbeads. It should be noted that the expression of EGFP was not completely turned off by removal of the microbeads when the protein synthesis process was carried out using a cell extract prepared from a RNase E-deficient *E.coli* strain (BL21(DE3)Star™). Instead, the EGFP fluorescence continued to increase with a slightly reduced rate even following the removal of the plasmid-conjugated magnetic beads. In a previous study, we showed that the functional stability of mRNA during cell-free protein synthesis is dramatically improved when a RNase E-deficient cell extract is employed [7]. Although enhanced mRNA stability leads to improved efficiencies for protein production when linear DNAs, such as PCR products, are used to direct the cell-free process, the work shows that delayed degradation of mRNA causes a sluggish response to the ON-to-OFF deprogramming of the reaction mixture. In contrast, an almost instant ON/OFF response was observed when protein synthesis employed a RNase E-containing BL21(DE3) extract (Figure 3).

### Sequential programming of the cell-free synthesis system with multiple target genes

**Figure 4:**
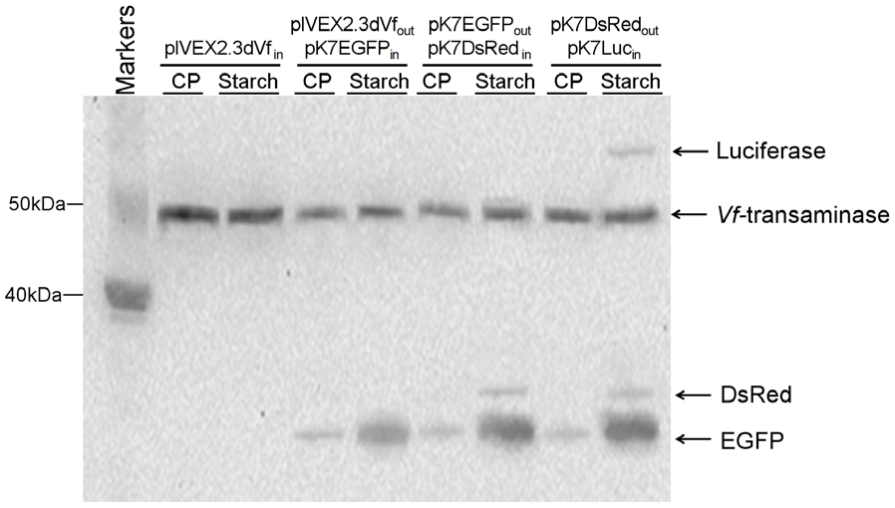
Sequential expression of multiple genes in cell-free synthesis systems employing different ATP regeneration methods. Western blot analysis of different reaction mixtures. Vf-transaminase-EGFP-DsRed-Luciferase were sequentially expressed in the reaction mixture using creatine phosphate or soluble starch as the energy source with a 90 min incubation time for each plasmid. Reaction samples were withdrawn at each point of the plasmid exchange.

The removability of the template plasmid also enables “resetting” the reaction mixture after expression of a single protein for subsequent “reprogramming” with a second target gene. This feature would enable sequential expression of multiple genes in the same reaction mixture. To probe this capability, we attempted to express in a serial fashion four different proteins through repeated removal and addition of the microbead-bound template plasmids. The experimental design employed the following plasmids conjugated on magnetic beads pIVEX2.3dVf, pK7EGFP, pK7DsRed, and pK7Luc. These plasmids, in the order given above, were sequentially incubated in the reaction mixture, removed using a neodymium magnet, and replaced by the next template plasmid. In the sequential expression of many protein species it would be desirable to have the reaction mixture support cell-free protein synthesis over extended time periods. However, ATP regeneration from creatine phosphate typically can be carried out for no longer than 2 h [8]. Therefore, when the incubation periods of individual templates were set to 90 min, the reaction mixture energized by creatine phosphate was only capable of producing the first and second proteins Vf-transaminase and EGFP (Figure 4). Recently, we described a long-living cell-free synthesis system that employs soluble starch as the energy reservoir. In this system, the duration of the protein synthesis reaction could be extended up to 10 h as a consequence of the slow and steady release of glucose into the reaction mixture, which leads to a continuous supply of ATP [9]. As expected, extended ATP supply with the use of soluble starch as an energy source allowed the successful expression of all four of the target proteins during the sequential expression experiment (Figure 4).

### Adjusting the relative expression levels of multiple genes during sequential expression

The efficiency of gene expression is affected by numerous factors including codon usage [10], [11], mRNA secondary structure [12] and nucleotide sequences in downstream regions [13], [14]. Therefore, it was not surprising to observe that different levels of expression occurred between the magnetic bead conjugate promoted, sequentially expressed proteins (Figure 4). No simple ways exist to control in a precise manner the relative expression levels of multiple genes in typical cell-free protein synthesis systems as well as in living cells. However, the process involving immobilization of template DNA on the removable microbeads does possess the flexibility to adjust the time period used for individual protein synthesis reactions. As a result, accumulation levels of various proteins can be controlled by using different incubation periods for each plasmid.

**Figure 5:**
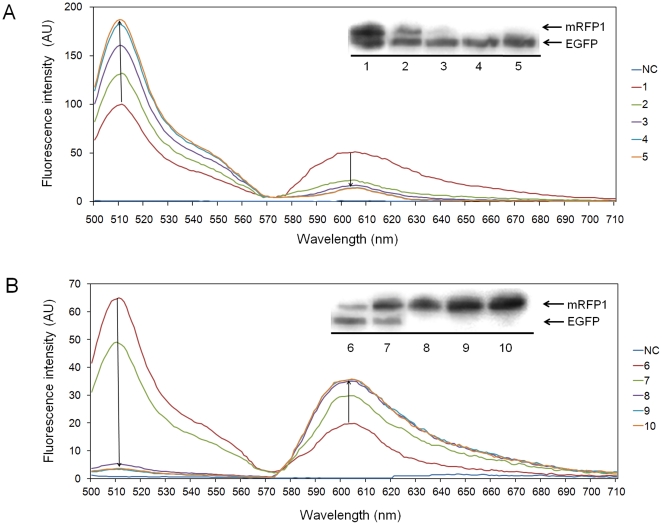
Tuning relative expression levels of EGFP and mRFP during sequential expression. Western blot and fluorescence analysis results from monitoring changes in the relative expression levels of the target proteins. NC represents background signal from the reaction that does not contain any template DNA. Magnetic microbead-conjugated plasmids pK7 EGFP and pK7 mRFP were sequentially incubated in the cell-free synthesis reaction mixture using soluble starch as the energy source. With a total incubation time fixed at 180 min, the first template plasmid conjugated on the magnetic microbead was incubated for increasing incubation time periods of 30 min from 30 min to 150 min, followed by incubation of the second plasmid for time period increments of 30 min from 150 min to 30 min. Samples 1 to 5: sequential expression of EGFP and mRFP1, samples 6 to 10: sequential expression of mRFP1 and EGFP.

Two fluorescent proteins (EGFP and mRFP1) were selected to demonstrate the relative expression level controllability that is possible using the magnetic bead conjugate promoted cell-free synthesis reactions. Employing a total reaction time period of 3 h, the magnetic bead-conjugated plasmid pK7EGFP was expressed for various time periods before being replaced by the second pK7mRFP1 conjugated magnetic beads. As the Western blot and fluorescence analyses results summarized in Figure 5 show, the relative expression levels of EGFP and mRFP1 could be varied by simply adjusting the relative time periods used in the incubation processes.

**Figure 6:**
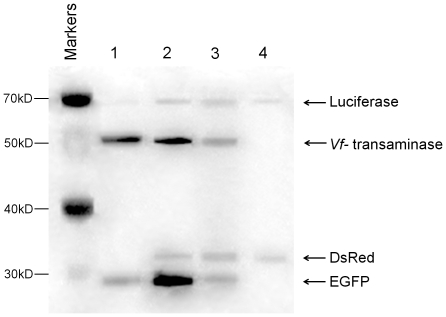
Relative expression levels of sequentially expressed proteins and the sequential expression and expression level control of multiple genes. Western blot analysis of reaction mixtures. Lane1: co-expresison of multiple genes. Lane2: Sequential expression of each of four different genes for 90 min. Lane3: The reaction times used for expression of DsRed and luciferase were 120 min and for Vf-transaminase and EGFP were 60 min. Lane4: Reaction times for expression of DsRed and luciferase were 150 min and for Vf-transaminase and EGFP were 30 min.

This technique was then applied to the control the relative expression levels of multiple proteins during the sequential expression of four plasmids (pIVEX2.3dVf, pK7EGFP, pK7DsRed, pK7Luc) in a reaction mixture with starch as the energy source. As demonstrated by the Western blot image shown in the first lane in Figure 6, co-expression of the four genes in the same reaction mixture led to different expression levels of the four target proteins, biased in favor of formation of the Vf-transaminase and EGFP. Only the expressions of the Vf-transaminase and EGFP were confirmed with clear bands, and the luciferase and DsRed were almost not detectable. Although the expressions of luciferase and DsRed were slightly recovered, overall, similar results were obtained when the four plasmids conjugated on magnetic beads were expressed sequentially (90 min each). We attempted to adjust the expression of the target proteins to similar levels by controlling the relative lengths of incubation of the magnetic microbead-conjugated plasmids. As shown in lane 3 of Figure 6, extended incubation periods of the plasmids pK7Luc and pK7DsRed led to increased expression levels of corresponding proteins, while the relative amounts of the Vf-transaminase and EGFP were reduced by shortened incubation of the plasmids. As a result, all of the target proteins could be produced in comparable levels. Further extension of the relative incubation periods of luciferase and DsRed led to a reversed bias towards their dominant expression (lane 4, Figure 6). To summarize, we demonstrate in this work the application of a cell-free protein synthesis system as a flexible platform for reversible programming of protein expression. ON/OFF control of gene expression was readily achieved through repeated addition and removal of bead-immobilized plasmid. The use of removable DNA template also enabled us to design the sequential order and relative expression levels of individual genes during the synthesis of multiple proteins. We expect that the presented method could be readily adapted to various areas including the production of important biomolecules, diagnostic and *in vitro* metabolic engineering.

## Materials and Methods

### Materials

Nucleotide triphosphates, creatine phosphate (CP) and creatine kinase (CK) were purchased from Roche Applied Science (Indianapolis, IN). Psoralen-biotin was from Pierce Chemicals (Rockford, IL). *E.coli* stains BL21(DE3) and BL21(DE3)-Star™ and streptavidin-coated magnetic beads were purchased from Invitrogen (Carlsbad, CA). All other reagents were purchased from Sigma (St Louis, MO) and used without purifications. The S12 extracts from the *E.coli* strains were prepared using the prviously described method [15]. Instead of using exogenous addition, the T7 RNA polymerase was expressed during the cultivation of the *E. coli* strains by induction with 1 mM isopropyl-D-thiogalactoside (IPTG) at 0.5 OD_600_. The cells were harvested 2 h after induction at 3.5 OD_600_ and processed to the S12 extracts. The plasmids pK7EGFP, pK7mRFP1, pK7DsRed, pK7Luc, pIVEX2.3d*Vf* that encode enhanced green fluorescent protein (EGFP), monomeric red fluorescent protein (mRFP1), red fluorescent protein derived from *Discosoma striata* (DsRed), firefly luciferase and ω-transaminase derived from *Vibrio fluvialis* (Vf-transaminase) under the control of the T7 promoter were used as the templates for protein synthesis.

### Biotinylation and immobilization of template plasmids

Template plasmids were immobilized onto streptavidin-coated magnetic beads through biotin-mediated conjugation [16]. Each plasmid (10 µg) dissolved in 100 µL of TE buffer (10 mM Tris, 1 mM EDTA, pH 7.4) was denatured at 90°C for 10 min and then quickly moved to −80°C. After 20 min, the plasmid solution was thawed and mixed with psoralen-biotin at different molar ratios to DNA (0.37, 3.7, 37, 370, 740, 1850, 3700). The mixtures were then irradiated with 365 nm UV light for 30 min so that the psoralen group of psoralen-biotin can be cross-linked with adenine and thymine residues in DNA [17]. Biotinylated plasmids were recovered by using ethanol precipitation and dissolved in distilled water.

Streptavidin-coated magnetic microbeads (250 µg) were washed three times and equilibrated in 125 µL of Binding/Wash buffer (5 mM Tris-HCl-pH 7.5, 0.5 mM EDTA, 1 M NaCl). The microbead suspension was then mixed with an equal volume of biotinylated plasmid solution dissolved in distilled water. After standing at room temperature for 2 h with gentle agitation, DNA-conjugated microbeads were recovered with a neodymium magnet, washed and resuspended in 125 µL of distilled water.

### Cell-free expression of bead-conjugated plasmids

Two types of cell-free synthesis systems, employing different ATP regeneration methods, were used in this study. For reactions using creatine phosphate as the energy source, the reaction mixture for protein synthesis was prepared with the following components in final volume of 30 µL: 57 mM HEPES-KOH (pH 7.5), 1.2 mM ATP, 0.85 mM each GTP, UTP, and CTP, 80 mM ammonium acetate, 12 mM magnesium acetate, 90 mM potassium glutamate, 34 µg/mL 1–5-formyl-5,6,7,8-tetrahydrofolic acid (folinic acid), 2.0 mM each of 20 amino acids, 2% polyethylene glycol 8000 (PEG8000), 0.3 U/mL creatine kinase, 67 mM creatine phosphate, and 8 µL of the S12 extract. In other reactions employing extended time periods, creatine phosphate and creatin kinase were replaced by 3.2% (w/v) soluble starch and 20 mM potassium phosphate as described in a previous report [9]. After being loaded with 250 µg of the bead-conjugated plasmid prepared as described above, the reaction mixture was incubated for 3 h with gentle agitation at 30°C. Accumulation of EGFP in the reaction mixture was monitored by measuring the fluorescence in a multiwell fluorescence reader (VICTOR™ X2, PerkinElmer, Waltham, MA) and Varian Cary Eclipse fluorescence spectrophotometer (Agilent technologies, Santa Clara, CA) with 485 nm and 509 nm excitation and emission wavelengths, respectively. Synthesis of mRFP1 in the reaction mixture was monitored by using 584 nm and 607 nm excitation and emission wavelengths, respectively. When necessary, emissions from EGFP and mRFP1 were scanned from 500 to 800 nm. In the experiments targeted at repeated or sequential expression of bead-conjugated plasmids, the magnetic beads were recovered using a neodymium magnet, washed twice with distilled water and reloaded into the next batches of protein synthesis reactions.

### SDS-PAGE and Western blot analyses of cell-free synthesized proteins

Cell-free synthesized proteins were analyzed by Coomassie Blue staining after running the reaction samples on a 12% Tricine–SDS–polyacrylamide gel as described by Schagger [18]. For Western blot analysis, proteins were transferred to PVDF membrane using a electrotransfer apparatus (Bio-Rad, Hercules, CA). The membrane was incubated in 1% casein blocking solution (1% casein, 200 mM Tris-Cl, 150 mM NaCl, pH 7.5) for 1 h at room temperature. The blocked membrane was then sequentially incubated with mouse monoclonal anti-polyhistidine antibody (Sigma, St Louis, MO) and alkaline phosphatase-conjugated goat anti mouse IgG antibody (Promega, Madison, WI) for 1 h at room temperature. Subsequently, signals from the secondary antibody were developed using a chemiluminescent HRP substrate (Millipore, Bedford, MA). After each step, the membrane was washed 1×15 min and 3×5 min with TBS-T buffer (50 mM Tris-Cl, 600 mM NaCl, 0.1% Tween 20, pH 7.6). The blotting images were obtained using ChemiDoc™ (Bio-Rad, Hercules, CA).
